# Implementing measurement error models with mechanistic mathematical models in a likelihood-based framework for estimation, identifiability analysis and prediction in the life sciences

**DOI:** 10.1098/rsif.2023.0402

**Published:** 2024-01-31

**Authors:** Ryan J. Murphy, Oliver J. Maclaren, Matthew J. Simpson

**Affiliations:** ^1^ School of Mathematics and Statistics, The University of Melbourne, Parkville, Victoria, Australia; ^2^ Department of Engineering Science and Biomedical Engineering, University of Auckland, Auckland, New Zealand; ^3^ Mathematical Sciences, Queensland University of Technology, Brisbane, Australia

**Keywords:** mathematical biology, systems biology, ordinary differential equations, partial differential equations, profile likelihood analysis, practical identifiability

## Abstract

Throughout the life sciences, we routinely seek to interpret measurements and observations using parametrized mechanistic mathematical models. A fundamental and often overlooked choice in this approach involves relating the solution of a mathematical model with noisy and incomplete measurement data. This is often achieved by assuming that the data are noisy measurements of the solution of a deterministic mathematical model, and that measurement errors are additive and normally distributed. While this assumption of additive Gaussian noise is extremely common and simple to implement and interpret, it is often unjustified and can lead to poor parameter estimates and non-physical predictions. One way to overcome this challenge is to implement a different measurement error model. In this review, we demonstrate how to implement a range of measurement error models in a likelihood-based framework for estimation, identifiability analysis and prediction, called profile-wise analysis. This frequentist approach to uncertainty quantification for mechanistic models leverages the profile likelihood for targeting parameters and understanding their influence on predictions. Case studies, motivated by simple caricature models routinely used in systems biology and mathematical biology literature, illustrate how the same ideas apply to different types of mathematical models. Open-source Julia code to reproduce results is available on GitHub.

## 1. Introduction

Mechanistic mathematical modelling and statistical uncertainty quantification are powerful tools for interpreting noisy incomplete data and facilitate decision making across a wide range of applications in the life sciences. Interpreting such data using mathematical models involves many different types of modelling choices, each of which can impact results and their interpretation. One of the simplest examples of connecting a mathematical model to data involves the use of a straight line model. A common approach to estimate a best-fit straight line involves linear regression and the method of ordinary least squares [1–4]. In this example, the mathematical model is chosen to be a straight line, *y* = *mx* + *c*, and the noisy data are assumed to be normally distributed with zero mean and constant positive variance about the true straight line. This assumption of additive Gaussian noise is a modelling choice that we refer to as an additive Gaussian measurement error model. Measurement error models are primarily used to describe uncertainties in the measurement process, and to a lesser extent random intrinsic variation [5]. Other similar terminologies include noise model, error model and observation error model, but here we will refer to this as a measurement error model. Here and throughout, we assume that measurement errors are uncorrelated, independent and identically distributed. Ordinary least-squares best-fit model parameters, $\hat{m}$ and $\hat{c}$, are estimated by minimizing the sum of the squared residuals, $E(m,c)=\sum_{i=1}^{I}{\left(y_{i}^{o}-y_{i}\right)}^{2}$, where the *i*th residual, for *i* = 1, 2, …, *I*, is the distance in the *y*-direction between the *i*th data point, $y_{i}^{o}$, and the corresponding point on the best-fit straight line, *y*_*i*_. Hence the name method of least squares. The best-fit straight line is then the mathematical model evaluated at the best-fit model parameters, i.e. $y=\hat{m}x+\hat{c}$, where $\hat{m}$ and $\hat{c}$ are the values of the slope and intercept that minimize *E*(*m*, *c*). Uncertainty in this example can be captured through the use of confidence intervals for model parameters, a confidence interval for the straight line based on the uncertainty in the model parameters, and a prediction interval for future observations [1–4].

In this review, we present a general framework extending these concepts to mechanistic mathematical models, in the form of systems of ordinary differential equations (ODEs) and systems of partial differential equations (PDEs), that are often considered in the systems biology literature and the mathematical biology literature, respectively. In particular, our primary focus is on the fundamental question of how to connect the output of a mathematical model to data using a variety of measurement error models.

The additive Gaussian measurement error model is ubiquitous and simple to interpret for mechanistic mathematical models, and often relates to estimating a best-fit model solution using nonlinear regression and a least-squares estimation problem [6,7]. Nonlinear regression extends the concept of linear regression to models where there is a nonlinear dependence between model parameters and model outputs that is typical for many deterministic ODEs and PDEs. Use of an additive Gaussian error model is often justified via the central limit theorem. However, the assumption of additive Gaussian noise is often unjustified in practice and, as we demonstrate, this can have important consequences because this assumption can lead to poor parameter estimates and non-physical predictions. Furthermore, even when the additive Gaussian error model is a reasonable choice it may not always be the most appropriate. In general, there are many ways in which noise could impact a system. For example, multiplicative noise models are often thought to be more relevant to problems in some parts of the systems biology literature [8–14]. One approach to tackle such challenges is to implement a different measurement error model. Here, we present a practical guide to implement a variety of measurement error models. Then, using illustrative case studies, we explain how to interpret results. Our approach in this review is not to claim that one noise model is superior to another, but to illustrate how relatively straightforward it can be to implement different noise models with different types of mathematical models.

All modelling choices, including the choice of a relevant mechanistic mathematical model and the choice of how to connect the mathematical model to data, should be considered on a case-by-case basis. As our focus is on the implementation of different error measurement models for ease of exposition we choose to explore simple caricature mathematical models from the systems biology literature and the mathematical biology literature rather than focusing on very specific models that might be relevant to a smaller audience. The kinds of mathematical models we explore include systems biology-type systems of ODEs [15–17], mathematical biology-type systems of PDEs [18–22] and difference equations [20,21,23–26]. Mathematical models of greater complexity are straightforward to explore using the methods presented in this study and our open source software can be adapted to deal with more biologically complicated models as required. Measurement error models can take many forms, for example discrete, continuous, additive and multiplicative, and the framework is well suited to explore these different options. We do not preference any particular measurement error model; however, we do illustrate that the framework can be used to help distinguish between the suitability of different choices of error model, such as choosing an error model that ensures non-negative predictions for quantities like concentrations or population densities.

**Figure 1.**
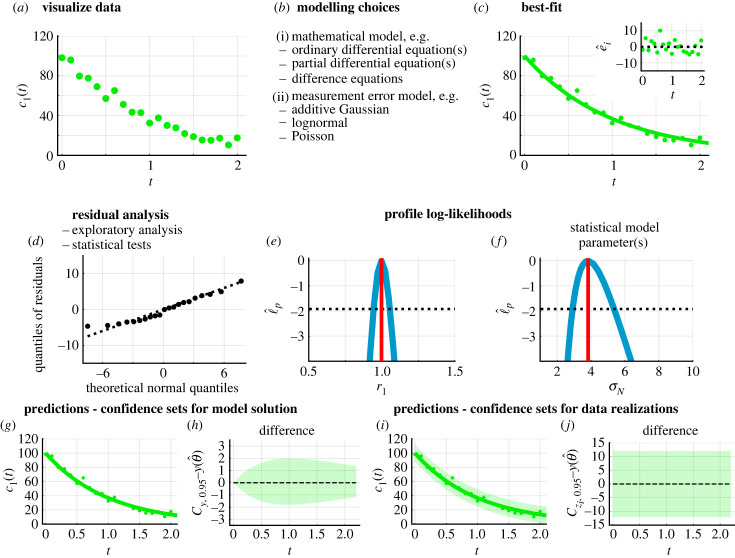
Implementing a variety of measurement error models in a profile likelihood-based framework for parameter estimation, identifiability analysis and prediction. (*a*) Synthetic data (circles). (*b*) The framework is applicable to a range of mathematical models and measurement error models. Schematics show results for a simple exponential decay ordinary differential model, d*c*_1_(*t*)/d*t* = −*r*_1_*c*_1_(*t*), the additive Gaussian measurement error model, known model parameters *θ* = (*r*_1_, *σ*_N_) = (1.0, 5.0), fixed initial condition *c*_1_(0) = 100.0, and observed data at 21 equally spaced time points from *t* = 0.0 to *t* = 2.0. (*c*) Mathematical model simulated with the MLE $\hat{\theta }=(r_{1},\sigma_{N})=(0.99,3.83)$ (solid line). Inset of (*c*) shows residuals ${\hat{e}}_{i}=y_{i}^{o}-y_{i}(\hat{\theta })$ with time, *t*. (*d*) Residual analysis can take many forms; results show a normal quantile–quantile plot of residuals. Profile log-likelihoods (blue) shown for (*e*) *r*_1_ and (*f*) *σ*_N_ with MLE (vertical red) and an approximate 95% confidence interval threshold (horizontal black-dashed). Predictions in the form of (*g*) union of profile-wise confidence sets for the model solution and (*i*) the union of profile-wise Bonferroni correction-based confidence sets for data realizations. (*g*,*i*) The mathematical model simulated with the MLE (solid), synthetic data (circles) and confidence sets (shaded regions). (*h*,*j*) To examine the confidence sets in detail we show the difference between the respective confidence sets and the mathematical model simulated with the MLE.

We now outline the profile-wise analysis (PWA) [27] approach to estimation, identifiability analysis, and prediction for a set of data that takes the form of a time series of chemical concentrations, as is often the case in applications in systems biology. Crucial first steps are to visualize the data (figure 1*a*) and to implement certain modelling choices such as choosing between a continuous ODE or discrete difference model (figure 1*b*). As always, the choice of mathematical model should be considered with respect to structural identifiability of its parameters [28–33]. Structural parameter non-identifiability means that there is a non-unique choice of model parameters that lead to the same model solution, and this can severely impede our ability to interpret results mechanistically since our ability to understand and interpret data mechanistically is often related to parameter estimation. For example, suppose one seeks to estimate two parameters *λ* and *D* but only the product *λD* is identifiable in the model [21,34,35]. In such a situation, we will be unable to estimate the value of the individual parameters irrespective of the number of measurements. Tools to assess structural identifiability of ODEs are reviewed in [36], including DAISY [37], GENSSI2 [38] and the StructuralIdentifiability Julia package [39].

*Parameter estimation for the mathematical model and measurement error model.* Given a mathematical model and a measurement error model, we generate a best-fit model solution, analogous to a best-fit curve (figure 1*c*). To estimate the best-fit model solution, we work within a likelihood-based framework. The likelihood function, *L*(*θ*|*D*), is related to the probability of observing data *D* as a function of the parameters *θ* [40]. In this setting, the best-fit model solution corresponds to the output of the mathematical model simulated at the model parameters which are found to be ‘best’ in the sense of those parameters that maximize *L*(*θ*|*D*). Parameters can be used to describe the mathematical model, such as *m* and *c* in the straight line example, and the noise, such as the variance $\sigma_{N}^{2}$ in the additive Gaussian measurement error model. In this work, we estimate both mathematical model parameters and statistical noise parameters simultaneously. Comparing the best-fit model solution with the data, and analysing residuals helps us to understand whether modelling choices are appropriate (figure 1*d*). Techniques to analyse standard additive residuals are reviewed in [6,7].

*Practical parameter identifiability.* While point estimates of best-fit model parameters are insightful, we often seek to understand how well parameters can be identified given a finite set of noisy incomplete data [16,27,31,41]. This question of practical parameter identifiability, and the subsequent components of the framework, can be explored using frequentist [16,27,31,40] or Bayesian methods [42–47]. While both approaches are generally interested in uncertainty quantification, we choose to work with a frequentist profile likelihood-based method that employs numerical optimization procedures [16,27,31,40,48–51]. The optimization procedures tend to be more computationally efficient than sampling-based methods for problems considered in this study [27,52,53]. We also choose to work with a frequentist framework since there are many estimation, identifiability and prediction workflows in Bayesian frameworks, but corresponding frequentist workflows that include prediction have received much less attention. Similarities and differences between our frequentist PWA workflow and Bayesian workflows are explored in [27]. While working with a full likelihood-based approach is relatively straightforward for models with a small number of parameters, this approach becomes computationally challenging for more complicated models with many parameters. By using a profile likelihood-based method, we can target individual parameters of interest, explore their practical identifiability, and form approximate confidence intervals (figure 1*e*,*f*) [40].

*Prediction.* Given a set of estimated model parameters, together with an estimate of the uncertainty in our estimates, it is natural to seek to understand how uncertainty in model parameters impacts predictions of model solutions (mathematical model trajectories) and data realizations (unobserved measurements). This is important because practitioners are most likely to be interested in understanding the variability in predictions rather than variability in parameter estimates. In this framework, we show that using parameter estimates to generate predictions is a powerful tool to assess the appropriateness of modelling choices and to interpret results. Predictions in the form of profile-wise confidence sets for model solutions are introduced in [27,53,54] and allow for predictions at a finer resolution than the data (figure 1*g*,*h*). These methods are simpler to implement and interpret in comparison to previous prediction methods that can involve additional constrained optimization problems or integration based techniques [16,55–59]. An approach to form likelihood-based confidence sets for model realizations, where the model is composed of a mechanistic mathematical model and a measurement error model, was introduced in [27] and here we present concrete examples (figure 1*i*,*j*). We also demonstrate how to assess statistical coverage properties that are often of interest, including curvewise and pointwise coverage properties for predictions, and make comparisons to a gold-standard full likelihood-based approach [27].

This review is structured as follows. In §2, we detail how to implement different measurement error models for parameter estimation, identifiability analysis and prediction using profile likelihood-based techniques. In §3, we demonstrate the generality of the framework by exploring a variety of measurement error models using illustrative case studies motivated by systems biology-type models and mathematical biology-type models. In §4, we present an explicit example of how to evaluate statistical coverage properties. Electronic supplementary material presents additional results including a comparison to a full likelihood-based approach [27]. To aid with understanding and reproducibility, all open source Julia code used to generate results is freely available on GitHub (https://github.com/ryanmurphy42/Murphy2023ErrorModels).

## 2. Parameter estimation, identifiability analysis and prediction

Here, we detail the PWA profile likelihood-based framework for parameter estimation, identifiability analysis and prediction. Throughout, we assume that experimental measurements are noisy observations of a deterministic mechanistic mathematical model. This framework is very general as it applies to cases where measurement error models may be additive, multiplicative, discrete, or continuous. As illustrative examples, we explicitly discuss and implement additive Gaussian noise, multiplicative lognormal and Poisson noise models. Mechanistic mathematical models may take many forms, for example systems of ODEs, systems of PDEs, and systems of difference equations. We choose to work with simple models to focus on the implementation of the framework and to make this work of interest to the broadest possible audience, as opposed to focusing on the details of specific mathematical models that are likely to be of interest to a smaller community. Our hope is that by focusing on fundamental mathematical models and providing open source code, readers can adapt these ideas to suit specific models for their particular area of interest.

### 2.1. Data

We consider temporal data that are often reported in the systems biology literature and are often interpreted in terms of models of chemical reaction networks and gene regulatory networks, and spatio-temporal data that are often reported in mathematical biology literature and interpreted using reaction–diffusion models. Temporal data are recorded at specified times. Spatio-temporal data are recorded at specified times and spatial positions. We let $y_{i}^{o}$ denote the *i*th experimental measurement at time *t*_*i*_ and spatial position *x*_*i*_. The superscript ‘o’ is used to distinguish the observed data from mechanistic mathematical model predictions. The spatial position, *x*_*i*_, may be a scalar or vector, and is omitted for temporal data. We represent multiple measurements at the same time and spatial position using distinct subscript indices. Assuming *I* experimental measurements, we collect the individual noisy measurements into a vector $y_{1\;:\;I}^{o}$, collect the observation times into a vector *t*_1:*I*_, and, for spatio-temporal data, collect the spatial positions into a vector *x*_1:*I*_.

### 2.2. Mechanistic mathematical model

We consider a variety of temporal and spatio-temporal mechanistic mathematical models. Temporal models in systems biology often take the form of systems of ODEs [15–17]:

2.1 $$\frac{dy(t)}{dt}=f\;(y(t);\theta_{M}),$$

where *y*(*t*) = (*y*^(1)^(*t*), *y*^(2)^(*t*), …, *y*^(*n*)^(*t*)) represents an *n*-dimensional vector of model solutions at time *t*, and *θ*_M_ represents a vector of mathematical model parameters. Noise free mathematical model solutions are evaluated at each *t*_*i*_, denoted *y*_*i*_(*θ*_M_) = *y*(*t*_*i*_; *θ*_M_), and collected into a vector *y*_1:*I*_(*θ*_M_).

Spatio-temporal models often take the form of systems of PDEs. In mathematical biology, we often consider systems of advection–diffusion–reaction equations [18–22]:

2.2 $$\frac{\partial y(t,x)}{\partial t}=f\left(y(t,x),\frac{\partial y(t,x)}{\partial x},\frac{\partial^{2}y(t,x)}{\partial x^{2}};\theta_{M}\right),$$

where *y*(*t*, *x*) = (*y*^(1)^(*t*, *x*), *y*^(2)^(*t*, *x*), …, *y*^(*n*)^(*t*, *x*)) represents an *n*-dimensional vector of model solutions at time *t* and position *x*, and *θ*_M_ represents a vector of mathematical model parameters. Noise free mathematical model solutions, evaluated at *t*_*i*_ and *x*_*i*_ are denoted *y*_*i*_(*θ*_M_) = *y*(*t*_*i*_, *x*_*i*_; *θ*_M_), and collected into a vector *y*_1:*I*_(*θ*_M_). The framework is well suited to consider natural extensions of equation (2.2), for example additional mechanisms such as nonlinear diffusion or non-local diffusion or PDE models in higher dimensions or in different coordinate systems [20,21]. The framework is also well suited to consider many more mechanistic mathematical models, for example difference equations (electronic supplementary material, S4). In all such examples the noise free output of the mathematical model can be collected into a vector *y*_1:*I*_(*θ*_M_).

### 2.3. Measurement error models

Measurement error models are a powerful tool to describe and interpret the relationship between experimental measurements, $y_{i}^{o}$, and noise free mathematical model solutions, *y*_*i*_(*θ*_M_). We take the common approach and assume that experimental measurements are noisy observations of a deterministic mechanistic mathematical model. This often corresponds to uncorrelated, independent, and identically distributed additive errors or multiplicative errors, in which case measurement errors are of the form $e_{i}=y_{i}^{o}-y_{i}(\theta_{M})$ or $e_{i}=y_{i}^{o}/y_{i}(\theta_{M})$, respectively. Good agreement between the data and the solution of a mathematical model corresponds to *e*_*i*_ = 0 for additive errors and *e*_*i*_ = 1 for multiplicative noise. In practice, the true model solution *y*(*θ*_M_) is unknown and we use a prediction of the best-fit model solution $y(\hat{\theta })$. Therefore, for additive errors, we analyse standard additive residuals taking the form ${\hat{e}}_{i}=y_{i}^{o}-y_{i}(\hat{\theta })$. While it is common to analyse multiplicative noise via additive residuals in log-transformed variables, i.e. $\log(y_{i}^{o})-\log(y_{i}(\theta ))={\hat{e}}_{i}$ [11], here we take a more direct approach and analyse the ratio ${\hat{e}}_{i}=y_{i}^{o}/y_{i}(\hat{\theta })$. Error models can take many forms, including discrete or continuous models, and are typically characterized by a vector of parameters *θ*_E_. The full model, comprising the mathematical model and measurement error model, is then characterized by *θ* = (*θ*_M_, *θ*_E_). We will demonstrate that it is straightforward to implement a range of measurement error models using three illustrative examples.

#### 2.3.1. Additive Gaussian model

The additive Gaussian model is ubiquitous, simple to interpret, and captures random errors and measurement uncertainties in a wide range of applications. Measurement errors are assumed to be additive, independent, and normally distributed with zero mean and constant variance, $\sigma_{N}^{2}>0$. Therefore, experimental measurements, $y_{i}^{o}$, are assumed to be independent and normally distributed about the noise free model solution, *y*_*i*_(*θ*_M_):

2.3 $$y_{i}^{o}|\theta ∼N(y_{i}(\theta_{M}),\sigma_{N}^{2}).$$

Under this noise model, the mean, median and mode of the distribution of possible values of $y_{i}^{o}|\theta$ are identical and equal to *y*_*i*_(*θ*). The variance is $\sigma_{N}^{2}$ and *θ*_E_ = *σ*_*N*_. Using this error model to obtain a best-fit solution of the mathematical model to the data, in the form of a maximum-likelihood estimate, reduces to a nonlinear least-squares problem. However, this error model is not always appropriate. Data in systems and mathematical biology are often non-negative, for example chemical concentrations or population densities. Implementing the additive Gaussian error model for data close to zero can be problematic and lead to non-negative physically unrealistic predictions as we will explore later in several case studies.

#### 2.3.2. Lognormal model

The lognormal model is employed to ensure non-negative and right-skewed errors in a range of biological applications [10–14]. This error model is multiplicative and we write

2.4 $$y_{i}^{o}|\theta =y_{i}(\theta )\eta_{i},\;\mathrm{where}\;\eta_{i}∼\mathrm{LogNormal}(0,\sigma_{L}^{2}).$$

Here, *θ*_E_ = *σ*_*L*_ and *η*_*i*_ are assumed to be independent. Equation (2.4) can also be written as $y_{i}^{o}|\theta ∼\mathrm{LogNormal}$ $\left(\log(y_{i}(\theta )),\sigma_{L}^{2}\right)$. Key statistics for the distribution of possible values of $y_{i}^{o}|\theta$ include the mean $y_{i}(\theta )\exp(\sigma_{L}^{2}/2)$, median *y*_*i*_(*θ*), mode $y_{i}(\theta )\exp(-\sigma_{L}^{2})$ and variance ${\left(y_{i}(\theta )\right)}^{2}\exp(\sigma_{L}^{2})[\exp(\sigma_{L}^{2})-1]$. In contrast to the additive Gaussian model which has constant variability over time, with the lognormal model variability increases as *y*_*i*_(*θ*) increases and variability vanishes as *y*_*i*_(*θ*) → 0^+^. The lognormal error model can also be written as $y_{i}^{o}|\theta =y_{i}(\theta )\exp(\varepsilon_{i})$ where $\varepsilon_{i}∼N(0,\sigma_{L}^{2})$ and is equivalent to implementing an additive Gaussian error model for log-transformed experimental measurements and log-transformed noise free model solutions, i.e. $\log(y_{i}^{o})|\theta ∼N(\log(y_{i}(\theta_{M})),\sigma_{L}^{2})$.

#### 2.3.3. Poisson model

The Poisson model is commonly employed to analyse non-negative count data [23,27,60]. Unlike the previous two measurement error models, we do not introduce additional parameters to describe this error model, so *θ* = *θ*_M_, and we write

2.5 $$y_{i}^{o}|\theta ∼\mathrm{Pois}(y_{i}(\theta )).$$

The Poisson distribution in equation (2.5) is a discrete probability density function that is neither additive or multiplicative. The model is only appropriate when observed data, $y_{i}^{o}$, are non-negative integers. However, there are no such technical restrictions for the output of the mathematical model and *y*_*i*_(*θ*) may take any non-negative value. When *y*_*i*_(*θ*) = 0 we consider the limit of Poisson distribution such that the only possible outcome is $y_{i}^{o}=0$ [61]. Under the Poisson model key statistics for the distribution of possible values of $y_{i}^{o}|\theta$ include the mean *y*_*i*_(*θ*); the median lies between $\left\lfloor y_{i}(\theta )-1\right\rfloor$ and $\left\lfloor y_{i}(\theta )+1\right\rfloor$; the modes are *y*_*i*_(*θ*) and *y*_*i*_(*θ*) − 1 when *y*_*i*_(*θ*) is a positive integer and $\left\lfloor y_{i}(\theta )\right\rfloor$ when *y*_*i*_(*θ*) is a positive non-integer; and the variance is *y*_*i*_(*θ*) [62]. In contrast to the additive Gaussian model which has approximately constant variability over time, with the Poisson model variability increases as *y*_*i*_(*θ*) increases and variability vanishes as *y*_*i*_(*θ*) → 0^+^.

### 2.4. Parameter estimation

We perform parameter estimation for the full model that comprises two components: (i) a mechanistic mathematical model and (ii) a measurement error model. We take a general approach and simultaneously estimate the full model parameters *θ*. This means that we estimate the mathematical model parameters, *θ*_M_, and measurement error model parameters, *θ*_*E*_, simultaneously. It is straightforward to consider special cases of this approach where a subset of the full model parameters *θ* may be pre-specified or assumed known, for example in cases where the measurement error model parameters *θ*_*E*_ can be pre-specified [43,52].

Taking a likelihood-based approach to parameter estimation, we use the log-likelihood:

2.6 $$\ell (\theta |y_{1\;:\;I}^{o})=\sum_{i=1}^{I}\log[\phi (y_{i}^{o};y_{i}(\theta ),\theta )],$$

where $\phi (y_{i}^{o};y_{i}(\theta ),\theta )$ represents the probability density function related to the measurement error model. For the additive Gaussian error model $\phi (y_{i}^{o};y_{i}(\theta ),\theta )=$ $\hat{\phi }(y_{i}^{o};y_{i}(\theta ),\sigma_{N}^{2}(\theta ))$, where $\hat{\phi }(x;\mu ,\sigma^{2})$ represents the Gaussian probability density function with mean *μ* and variance *σ*^2^. For the lognormal error model $\phi (y_{i}^{o};y_{i}(\theta ),\theta )=$ $\hat{\phi }(y_{i}^{o};\log\left(y_{i}(\theta )\right),\sigma_{L}^{2}(\theta ))$, where $\hat{\phi }(x;\mu ,\sigma )$ represents the probability density function of the Lognormal(*μ*, *σ*^2^) distribution. For the Poisson error model, $\phi (y_{i}^{o};y_{i}(\theta ),\theta )=$ $\hat{\phi }(y_{i}^{o};y_{i}(\theta ))$, where $\hat{\phi }(x;\lambda )$ represents the probability density function for the Poisson distribution with rate parameter *λ*.

To obtain a point-estimate of *θ* that gives the best match to the data, in the sense of the highest likelihood, we seek the maximum-likelihood estimate (MLE):

2.7 $$\hat{\theta }=\underset{\theta }{\mathrm{argmax}}\;\ell (\theta |y_{1\;:\;I}^{o}).$$

We estimate $\hat{\theta }$, subject to bound constraints, using numerical optimization.

### 2.5. Identifiability analysis using the profile likelihood

We are often interested in the range of parameters that give a similar match to the data as the MLE. This is analogous to asking whether parameters can be uniquely identified given the data. There are two approaches to address this question of parameter identifiability: structural identifiability and practical identifiability. Structural identifiability explores whether parameters are uniquely identifiable given continuous noise free observations of model solutions. Many software tools, using symbolic calculations, have been developed to analyse structural identifiability for systems of ODEs as reviewed in [36]. Tools to assess structural identifiability of systems of PDEs have not been widely developed [63], and structural identifiability analysis of PDE models is an active area of research.

Practical identifiability assesses how well model parameters can be identified given a finite set of noisy incomplete data. To explore practical identifiability we use a profile likelihood-based approach and work with the normalized log-likelihood:

2.8 $$\hat{\ell }(\theta |y_{1\;:\;I}^{o})=\ell (\theta |y_{1\;:\;I}^{o})-\ell (\hat{\theta }|y_{1\;:\;I}^{o}).$$

Normalizing the log-likelihood means that $\hat{l}(\theta |y_{1\;:\;I}^{o})\leq 0$ and $\hat{l}(\hat{\theta }|y_{1\;:\;I}^{o})=0$.

To assess practical identifiability of parameters within the full parameter vector, *θ*, we partition *θ* as *θ* = (*ψ*, *λ*) where *ψ* can represent any combination of parameters and *λ* represents the complement [27,40,64,65]. In this section, we assess whether each parameter within the full parameter vector is practically identifiable in turn. We consider *ψ* to represent a scalar parameter of interest and *λ* to represent a vector of the remaining nuisance parameters. This allows us to focus on univariate profile likelihoods. We now work with the profile log-likelihood for the scalar interest parameter *ψ* [40,66]

2.9 $${\hat{\ell }}_{\;p}(\psi |y_{1\;:\;I}^{o})=\underset{\lambda |\psi }{\sup}\;\hat{\ell }(\psi ,\lambda |y_{1\;:\;I}^{o}),$$

where the subscript *p* is introduced to denote the profile log-likelihood. Therefore, the profile log-likelihood maximizes the normalized log-likelihood for each value of the scalar *ψ*. This process implicitly defines a function λ*(*ψ*) of optimal values of *λ* for each *ψ*, and defines a curve with points (*ψ*, λ*(*ψ*)) in parameter space that includes the MLE, $\hat{\theta }=(\hat{\psi },\hat{\lambda })$. To estimate ${\hat{\ell }}_{\;p}(\psi |y_{1\;:\;I}^{o}),$ we define a mesh of 2*N* points for *ψ* comprising *N* equally spaced points from a pre-specified lower bound, *ψ*_L_, to $\hat{\psi }$ and *N* equally spaced points from $\hat{\psi }$ to a pre-specified upper bound, *ψ*_U_. We choose the lower and upper bounds to capture approximate confidence intervals. We choose the number of mesh points so that there are many points within the approximate confidence interval; typically we choose *N* = 20. Further details on how the choice of *N* impacts coverage properties are presented in §2.6.1. For each value of *ψ* in the mesh, we estimate ${\hat{\ell }}_{\;p}(\psi |y_{1\;:\;I}^{o})$, subject to the bound constraints for *λ*, using numerical maximization.

Univariate profile log-likelihoods for scalar interest parameters, referred to as profiles for brevity, provide a visual and quantitative tool to assess practical identifiability. A narrow univariate profile that is well formed about a single peak corresponds to a parameter of interest that is practically identifiable, while a wide flat profile indicates that the parameter of interest is not practically identifiable. We assess narrow and wide relative to log-likelihood-based approximate confidence intervals. We define the log-likelihood-based approximate confidence interval for the scalar *ψ* from the profile log-likelihood:

2.10 $$C_{\psi ,1-\alpha }(y_{1\;:\;I}^{o})=\{\psi |{\hat{\ell }}_{\;p}(\psi |y_{1\;:\;I}^{o})\geq \ell_{c}\},$$

where the threshold parameter ℓ_*c*_ is chosen such that the confidence interval has an approximate asymptotic coverage probability of 1 − *α*. Many studies report 90%, 95%, 99% or 99.9% confidence intervals for univariate profiles [40,67]. These thresholds are calibrated using the *χ*^2^ distribution, which is reasonable for sufficiently regular problems [40,67]. In particular, ℓ_*c*_ = −Δ_*ν*,1−*α*_/2, where Δ_*ν*,1−*α*_ refers to the (1 − *α*) quantile of a *χ*^2^ distribution with *ν* degrees of freedom set equal to the dimension of the interest parameter, e.g. *ν* = 1 for univariate profiles. It is straightforward to extend this approach to consider vector-valued interest parameters, for example to generate bivariate profiles [27].

### 2.6. Predictions

We generate predictions for model solutions, *y* = *y*(*t*; *θ*), and data realizations, *z*_*i*_, using a profile log-likelihood-based approach. These predictions propagate forward uncertainties in interest parameters and allow us to understand and interpret the contribution of each model parameter, or unions of parameters, to uncertainties in predictions. This step is very important when using mathematical models to interpret data and to communicate with collaborators from other disciplines simply because predictions and variability in predictions are likely to be of greater interest than estimates of parameter values in a mathematical model.

#### 2.6.1. Confidence sets for deterministic model solutions

We now propagate forward uncertainty in a scalar interest parameter, *ψ*, to understand and interpret the uncertainty in predictions of the model solution, *y* = *y*(*t*; *θ*). The approximate profile-wise log-likelihood for the model solution, *y*, is obtained by taking the maximum profile log-likelihood value over all values of *ψ* consistent with *y*(*t*; (*ψ*, λ*(*ψ*))) = *y*, i.e.

2.11 $${\hat{\ell }}_{\;p}\left(y(t;(\psi ,\lambda^{*}(\psi )))=y|y_{1\;:\;I}^{o}\right)=\underset{\psi |y(t;(\psi ,\lambda^{*}(\psi )))=y}{\sup}\;{\hat{\ell }}_{\;p}(\psi |y_{1\;:\;I}^{o}).$$

Here, *y*(*t*; (*ψ*, λ*(*ψ*))) corresponds to the output or solution of the mechanistic mathematical model solved with parameter values *θ* = (*ψ*, λ*(*ψ*)). The confidence set for the model solution, *y*, propagated from the scalar interest parameter *ψ* is

2.12 $$C_{y,1-\alpha }^{\psi }(y_{1\;:\;I}^{o})=\{y\;|\;{\hat{\ell }}_{\;p}\left(y(t;(\psi ,\lambda^{*}(\psi )))=y\;|\;y_{1\;:\;I}^{o}\right)\geq \ell_{c}\}.$$

In practice, we form an approximate (1 − *α*)% confidence interval, $C_{y,1-\alpha }^{\psi }(y_{1\;:\;I}^{o})$, by simulating *y*(*t*; (*ψ*, λ*(*ψ*))) for each $\psi \in C_{\psi ,1-\alpha }(y_{1\;:\;I}^{o})$. This confidence set can be used to reveal the influence of uncertainty in *ψ* on predictions of the model solution. From an implementation perspective, this is where the number of mesh points used to compute profiles can be important and should be considered on a case-by-case basis. If there are not enough mesh points in the confidence interval then the confidence sets will not have good coverage properties. For example, in the extreme case of only one mesh point in the confidence interval the confidence set would only be the mathematical model simulated at the MLE and would not provide any insight into uncertainty.

Each parameter in *θ* can be treated in turn as an interest parameter. Therefore, for each parameter in *θ*, we can construct an approximate confidence interval $C_{y,1-\alpha }^{\psi }(y_{1\;:\;I}^{o})$. Comparing approximate confidence intervals constructed for different parameters in *θ* illustrates which parameters contribute to greater uncertainty in model solutions [54]. This can be important for understanding how to improve predictions and for experimental design. However, optimizing out nuisance parameters in this profile log-likelihood-based approach typically leads to lower coverage than other methods that consider all uncertainties simultaneously, especially when the model solution has weak dependence on the interest parameter and non-trivial dependence on the nuisance parameters [53]. More conservative approximate confidence sets, relative to the individual profile-wise confidence sets, can be constructed by taking the union of individual profile-wise confidence sets for the model solution:

2.13 $$C_{y,1-\alpha }(y_{1\;:\;I}^{o})\approx \underset{\psi }{⋃}C_{y,1-\alpha }^{\psi }(y_{1\;:\;I}^{o}).$$

Equation (2.13) provides insight into the uncertainty due to all model parameters across the solution of the mathematical model. As we will demonstrate, this approach is a simple, computationally efficient and an intuitive model diagnostic tool. Furthermore, the method can be repeated with vector-valued interest parameters and increasing the dimension results in closer agreement to full likelihood-based methods [27]. As an example, the union of profile-wise confidence sets for two-dimensional interest parameters can be constructed by considering bivariate profiles for all pairs of parameters [27]. This approach can also be generalized beyond that of predictions of the model solution to predictions of data distribution parameters [27]. Note that for the additive Gaussian and Poisson measurement error models the model solution is the mean of the data distribution and for the lognormal measurement error model the model solution is the median of data distribution. These methods are simpler to implement and interpret in comparison to previous methods, such as those that involve additional constrained optimization problems [55–58].

#### 2.6.2. Confidence sets for noisy data realizations

In practice, we are often interested in using mathematical models to generate predictions of noisy data realizations, since an individual experiment measurement can be thought of as a noisy data realization. These predictions allow us to explore what we would expect to observe if we were to repeat the experiment or if we were to measure at different times and/or spatial positions. By building our framework on parametrized mechanistic mathematical models, we can also predict beyond the data based on a mechanistic understanding. In contrast to confidence sets for deterministic model solutions where it is natural to consider continuous trajectories, data are naturally defined at discrete time points; therefore here we consider confidence sets for noisy single time observations.

To form approximate (1−α)% confidence sets for model realizations, we consider a number of approaches: (i) a simple MLE-based approach that may not reach the desired coverage level and (ii) Bonferroni correction-based approaches that are likely to exceed the desired coverage level. To explain these approaches consider the problem of forming a (1−α)% confidence set for a single unknown data realization *z*_*i*_ at time *t*_*i*_ for *i* = 1, 2, …, *J*, where the variable *z*_*i*_ is used to distinguish the unknown data realization from an observed data realization $y_{i}^{o}$ at time *t*_*i*_. These predictions can be made at the same time points as observed data and can also be made at time points where observed data are not collected. In this review, to visualize the uncertainty throughout time, we generate predictions at a higher temporal resolution in comparison to the observed data. If the mathematical model, mathematical model parameters, measurement error model, and measurement error model parameters are all known then it is straightforward to form a confidence set for each *z*_*i*_. The bounds of the (1−α)% confidence set are obtained by computing the *α*/2 and 1 − *α*/2 quantiles of the probability distribution associated with the measurement error model and mathematical model solution at time *t*_*i*_. This procedure can be repeated for each unknown data realization at each time *t*_*i*_. For example, consider a scalar valued model solution, *y*, that depends only on time, with an additive Gaussian measurement error model where *σ*_*N*_ is known. The lower and upper bounds of the prediction set can be estimated at each point in time *t*_*i*_ by calculating the *α*/2 and 1 − *α*/2 quantiles of the normal distribution with mean *y*(*t*_*i*_) and standard deviation *σ*_*N*_. This computational approach naturally extends to other measurement error models, including the Poisson and lognormal models. In practice however, we typically face a more challenging scenario where the true model parameters and true mathematical model solution, *y* = *y*(*t*; *θ*), are all unknown, and we now outline two approaches for dealing with this situation.

*MLE-based approach.* When the true model parameters and true mathematical model solution are unknown a simple approach is to assume that the model parameters are given by the MLE, $\hat{\theta }$, and the true solution of the mathematical model is given by evaluating the solution of the model at the MLE, $y(t;\hat{\theta })$. With this assumption, it is then straightforward to generate a (1−α)% confidence set as previously described. In practice, it is unlikely that the MLE, $\hat{\theta }$, will be identical to the true model parameters, *θ*, so this approach may not reach the desired coverage level. However, when uncertainty due to statistical noise is large relative to the difference between *y*(*t*; *θ*) and $y(t;\hat{\theta })$ this simple MLE-based approach can work well.

*Bonferroni correction-based approaches.* A more conservative approach for forming confidence sets for model realizations involves propagating forward uncertainty in model parameters. The following approach was introduced in [27], and here we present concrete examples. Consider a scalar interest parameter *ψ* and a corresponding confidence set for the model solution, $C_{y,1-\alpha /2}^{\psi }(y_{1\;:\;I}^{o})$. For each $y\in C_{y,1-\alpha /2}^{\psi }(y_{1\;:\;I}^{o}),$ we construct a prediction set $A_{y,1-\alpha /2}^{\psi }(y_{1\;:\;I}^{o})$ such that the probability of observing a measurement $z_{i}\in A_{y,1-\alpha /2}^{\psi }(y_{1\;:\;I}^{o})$ is 1 − *α*/2. Computationally, $A_{y,1-\alpha /2}^{\psi }(y_{1\;:\;I}^{o})$ can be constructed in a pointwise manner by estimating the *α*/4 and 1 − *α*/4 quantiles of the probability distribution associated with the measurement error model. Taking the union for each $y\in C_{y,1-\alpha /2}^{\psi }(y_{i\;:\;I}^{o}),$ we obtain a conservative (1−α)% confidence set for model realizations from the interest parameter *ψ*:

2.14 $$C_{z_{i},1-\alpha }^{\psi }(y_{1\;:\;I}^{o})=\underset{y\in C_{y,1-\alpha /2}^{\psi }(y_{1\;:\;I}^{o})}{⋃}A_{y,1-\alpha /2}^{\psi }(y_{1\;:\;I}^{o}).$$

This approach employs a Bonferroni correction method [27,68].

Equation (2.14) represents a conservative confidence set for the data realizations *z*_*i*_ at the level of the individual interest parameter *ψ*. Treating each parameter in *θ* in turn as an interest parameter and taking the union results in a confidence set for the overall uncertainty in data realizations:

2.15 $$C_{z_{i},1-\alpha }(y_{1\;:\;I}^{o})\approx \underset{\psi }{⋃}C_{z_{i},1-\alpha }^{\psi }(y_{1\;:\;I}^{o}).$$

### 2.7. Coverage properties

Coverage properties of confidence intervals and confidence sets are defined formally, but for likelihood-based confidence sets coverage properties are expected to only hold asymptotically in data size. In practice, we can evaluate approximate statistical coverage properties numerically by repeated sampling. In particular, we can generate, and then analyse, many datasets using the same mathematical model, measurement error model, and true model parameters, *θ*. A detailed illustrative example for temporal data is discussed in §4. The procedure is applicable to a range of models and data.

## 3. Case studies

We will now implement the general framework using simple caricature mathematical models routinely used in the systems biology literature and the mathematical biology literature. The full models are formed by (i) a deterministic mathematical model and (ii) a measurement error model. Example mathematical models that we consider include systems of linear and nonlinear temporal ODEs often used in the systems biology literature and systems of spatio-temporal PDEs often used in the mathematical biology literature. Example measurement error models that we consider include additive Gaussian, lognormal and Poisson.

### 3.1. Temporal linear models

Consider a chemical reaction network with two chemical species *C*_1_ and *C*_2_. We assume that *C*_1_ decays to form *C*_2_ at a rate *r*_1_, and that *C*_2_ decays at a rate *r*_2_. Within this modelling framework, we do not explicitly model the decay products from the second reaction. Applying the law of mass action, the concentrations of *C*_1_ and *C*_2_ at time *t*, denoted *c*_1_(*t*) and *c*_2_(*t*), respectively, are governed by the following system of ODEs:

3.1 $$\begin{array}{rl} & \frac{dc_{1}(t)}{dt}=-r_{1}c_{1}(t)\\ \;\mathrm{and}\; & \frac{dc_{2}(t)}{dt}=r_{1}c_{1}(t)-r_{2}c_{2}(t). \end{array}\}$$

We refer to the terms on the right-hand side of equation (3.1) as the reaction terms, which are linear in this simple case. Equation (3.1) has an analytical solution, which for *r*_1_ ≠ *r*_2_ can be written as

3.2 $$\begin{array}{rl} & c_{1}(t)=c_{1}(0)\exp(-r_{1}t)\\ \;\mathrm{and}\; & c_{2}(t)=c_{1}(0)\exp(-r_{1}t)\left(\frac{r_{1}}{r_{2}-r_{1}}\right)+\left[c_{2}(0)-\frac{c_{1}(0)r_{1}}{r_{2}-r_{1}}\right]\exp(-r_{2}t). \end{array}\}$$

In the special case *r*_1_ = *r*_2_, we can write the exact solution in a different format where *c*_2_(*t*) is proportional to *c*_1_(*t*). We treat the initial conditions *c*_1_(0) and *c*_2_(0) as known so that equations (3.1)–(3.2) are characterized by two parameters *r*_1_ and *r*_2_ that we will estimate. Here, *r*_1_ and *r*_2_ are structurally identifiable. Initial conditions can also easily be treated as unknowns within this framework [54,69]. For parameter estimation, we solve equation (3.1) numerically which is convenient because we do not have to consider the cases *r*_1_ ≠ *r*_2_ and *r*_1_ = *r*_2_ separately in our numerical implementation.

**Figure 2.**
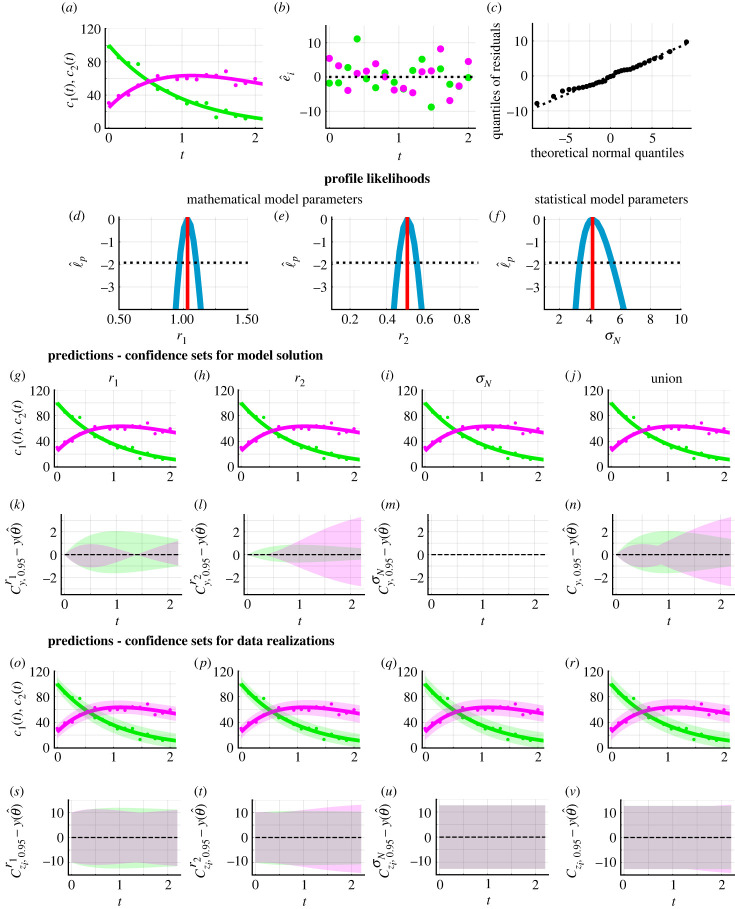
Caricature ODE model with linear reactions (equation (3.1)). (*a*) Synthetic data (circles) at 16 equally spaced time points from *t* = 0.0 to *t* = 2.0 are generated by simulating equation (3.1), the additive Gaussian measurement error model, known model parameters *θ* = (*r*_1_, *r*_2_, *σ*_N_) = (1.0, 0.5, 5.0), fixed initial conditions (*c*_1_(0), *c*_2_(0)) = (100.0, 25.0). The MLE, computed assuming an additive Gaussian measurement error model, is $\hat{\theta }=(r_{1},r_{2},\sigma_{L})=(1.03,0.51,4.18)$. Equation (3.1) simulated with the MLE (solid). Throughout *c*_1_(*t*) (solid green) and *c*_2_(*t*) (solid magenta). (*b*) Residuals ${\hat{e}}_{i}=y_{i}^{o}-y_{i}(\hat{\theta })$ with time *t*. (*c*) Normal quantile–quantile plot of residuals. (*d*–*f*) Profile log-likelihoods (blue) for (*d*) *r*_1_, (*e*) *r*_2_ and (*f*) *σ*_N_ with MLE (red-dashed), an approximate 95% confidence interval threshold (horizontal black-dashed). (*g*–*j*) Profile-wise confidence sets for the model solution (*g*) *r*_1_, (*h*) *r*_2_, (*i*) *σ*_N_ and (i) their union. (*k*–*n*) Difference between confidence set for model solution and the solution of the mathematical model evaluated at the MLE. (*o*–*r*) Profile-wise confidence sets for data realizations (shaded) for (*o*) *r*_1_, (*p*) *r*_2_, (*q*) *σ*_N_ and (*r*) their union. (*s*–*v*) Difference between confidence set for model realizations and the solution of the mathematical model evaluated at the MLE.

We now explore a simple example shown in figure 2 and specify (*c*_1_(0), *c*_2_(0)) = (100.0, 25.0). We generate synthetic data using equation (3.2), the additive Gaussian error model, and model parameters *θ* = (*r*_1_, *r*_2_, *σ*_N_) = (1.0, 0.5, 5.0) (figure 2*a*). Then, to demonstrate that the framework accurately recovers these known parameter values and to generate predictions, we use equation (3.2) and the additive Gaussian error model. Computing the MLE of the model parameters, we obtain $\hat{\theta }=(r_{1},r_{2},\sigma_{N})=(1.03,0.51,4.18)$. Simulating the deterministic mathematical model with MLE, we observe excellent agreement with the data (figure 2*a*). Inspecting the residuals, ${\hat{e}}_{i}=y_{i}^{o}-y_{i}(\hat{\theta })$, suggests that they appear, visually at least, to be independent and normally distributed (figure 2*b*). There are many techniques to analyse standard additive residuals in greater detail should a simple visual interpretation lead us to conclude that the residuals are not independent [6,7,51]. We take a simple and common graphical approach. We plot the residuals on a normal quantile–quantile plot (figure 2*c*). As the residuals appear close to the reference line on the normal quantile–quantile plot, the assumption of normally distributed residuals appears reasonable.

In practice, it is often crucial to understand whether model parameters can be approximately identified or whether many combinations of parameter values result in a similar fit to the data. To address this question of practical identifiability, we compute univariate profile log-likelihoods for *r*_1_, *r*_2_ and *σ*_N_. Each profile is well formed around a single central peak (figure 2*d*–*f*). This suggests that each model parameter is well identified by the data. Using the profile log-likelihoods, we compute approximate 95% confidence intervals, *r*_1_ ∈ (0.97, 1.10), *r*_2_ ∈ (0.45, 0.56) and *σ*_N_ ∈ (3.33, 5.46). These confidence intervals indicate the range of values for which we are 95% confident that the true values lie within. On this occasion, each component of the known parameter *θ* is contained within the respective confidence interval.

Thus far we have obtained estimates of best-fit parameters and associated uncertainties. To connect estimates of best-fit parameters and associated uncertainties to data, we need to understand how uncertainty in *θ* propagates forward to uncertainties in the dependent variables, here *c*_1_(*t*) and *c*_2_(*t*), as this is what is measured in reality. There are many predictions of *c*_1_(*t*) and *c*_2_(*t*) that one could make. We consider two key forms of predictions: confidence sets for deterministic model solutions and Bonferroni correction-based confidence sets for noisy data realizations. For each parameter, we generate confidence sets for the model solution and explore the difference between the confidence sets and the mathematical model simulated with the MLE (figure 2*g*–*n*). Results in figure 2*g*–*i*,*k*–*m* reveal the influence of individual model parameters on predictions of the model solution. For example, uncertainty in the parameter *r*_2_ corresponds to increasing uncertainty in the model solution for *c*_2_(*t*) as time increases, i.e. $C_{y,0.95}^{r_{2}}-y(\hat{\theta })$ increases with time for *c*_2_(*t*) (figure 2*h*,*l*). However, uncertainty in the measurement error model parameter, *σ*_*N*_, does not contribute to uncertainty in predictions of the model solution (figure 2*i*,*m*), since the noise is additive. Furthermore, we can observe that for *t* ≥ 1 uncertainty in *r*_2_ contributes to greater uncertainty in *c*_2_(*t*) than uncertainty in *r*_1_ (figure 2*g*,*h*,*k*,*l*). Predictions in the form of Bonferroni correction-based confidence sets for data realizations take into account the measurement error model (figure 2*o*–*v*). These can be generated for each individual parameter and an understanding of the overall uncertainty can be obtained by taking their union. Overall, results in figure 2 show that the framework recovers known parameter values and generates sensible predictions when the mathematical model and measurement error model are both known.

**Figure 3.**
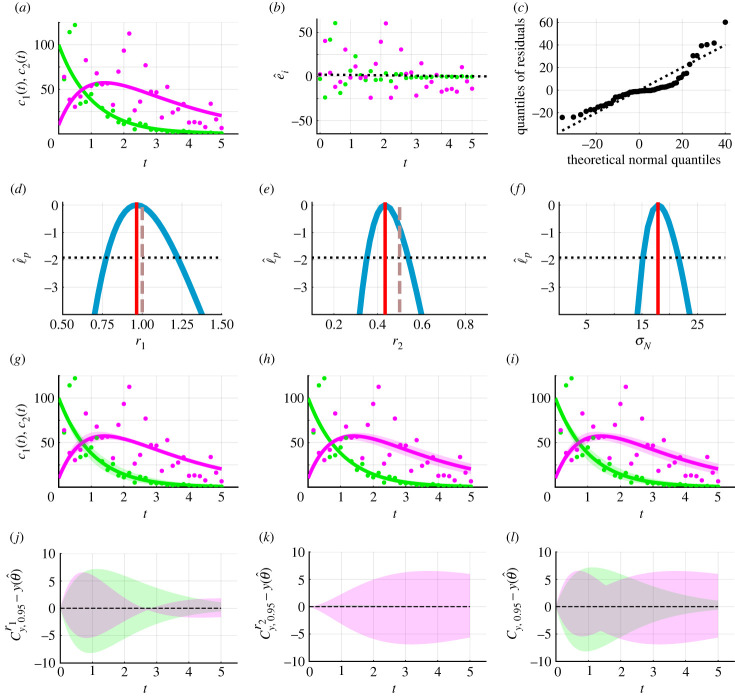
Caricature ODE model with linear reactions (equation (3.1)) and intentional misspecification of the measurement error model. (*a*) Synthetic data (circles) at 31 equally spaced time points from *t* = 0.0 to *t* = 5.0 are generated by simulating equation (3.1), the lognormal measurement error model, known model parameters *θ* = (*r*_1_, *r*_2_, *σ*_L_) = (1.0, 0.5, 0.4), and initial conditions (*c*_1_(0), *c*_2_(0)) = (100.0, 10.0). The MLE, computed assuming an additive Gaussian measurement error model, is $\hat{\theta }=(r_{1},r_{2},\sigma_{L})=(0.97,0.43,17.9)$. Equation (3.1) simulated with the MLE (solid). Throughout *c*_1_(*t*) (solid green) and *c*_2_(*t*) (solid magenta). (*b*) Residuals ${\hat{e}}_{i}=y_{i}^{o}-y_{i}(\hat{\theta })$ with time *t*. (*c*) Normal quantile–quantile plot of residuals. (*d*–*f*) Profile log-likelihoods (blue) for (*d*) *r*_1_, (*e*) *r*_2_ and (*f*) *σ*_N_ with MLE (red-dashed), an approximate 95% confidence interval threshold (horizontal black-dashed) and known model parameters (vertical brown dashed). (*g*–*i*) Profile-wise confidence sets for the model solution (*g*) *r*_1_, (*h*) *r*_2_ and (*i*) their union. (*j*,*k*,*l*) Difference between confidence set and the solution of the mathematical model evaluated at the MLE.

In practice faced with experimental data, we do not know which measurement model is appropriate. An extremely common approach in this situation is to assume an additive Gaussian measurement error model as we do in figure 2. This choice is simple to implement and interpret but the suitability of this choice is often unjustified. We now explore an example where assuming additive Gaussian errors is inappropriate and leads to physically unrealistic predictions. In figure 3*a*, we present synthetic data generated by simulating equation (3.2) and the lognormal error model with known parameter values, *θ* = (*r*_1_, *r*_2_, *σ*_L_) = (1.0, 0.5, 0.4), and initial conditions, (*c*_1_(0), *c*_2_(0)) = (100.0, 10.0). To estimate model parameters and generate predictions, we assume that the true mathematical model is known and intentionally misspecify the measurement error model.

Assuming an additive Gaussian error model, the MLE is $\hat{\theta }=(r_{1},r_{2},\sigma_{N})=(0.97,0.43,17.90)$. Evaluating equation (3.1) with the MLE we observe good agreement with the data (figure 3*a*). However, plotting the residuals, ${\hat{e}}_{i}=y_{i}^{o}-y_{i}(\hat{\theta })$, on a normal quantile–quantile plot shows a visually distinct deviation from the reference line with points representing the tails of the residuals above the reference line and points close the median of the residuals below the reference line (figure 3*c*). This suggests that the additive Gaussian measurement error model may be inappropriate. Nevertheless, we proceed with the additive Gaussian error model to demonstrate further issues that can arise and subsequent opportunities to detect the misspecified measurement error model. Profile log-likelihoods for *r*_1_, *r*_2_ and *σ*_N_ suggest that these parameters are practically identifiable and approximate 95% confidence intervals, *r*_1_ ∈ (0.77, 1.22) and *r*_2_ ∈ (0.35, 0.54), capture known parameter values. Due to the error model misspecification, we are unable to compare the approximate confidence interval for *σ*_N_ to a known value.

**Figure 4.**
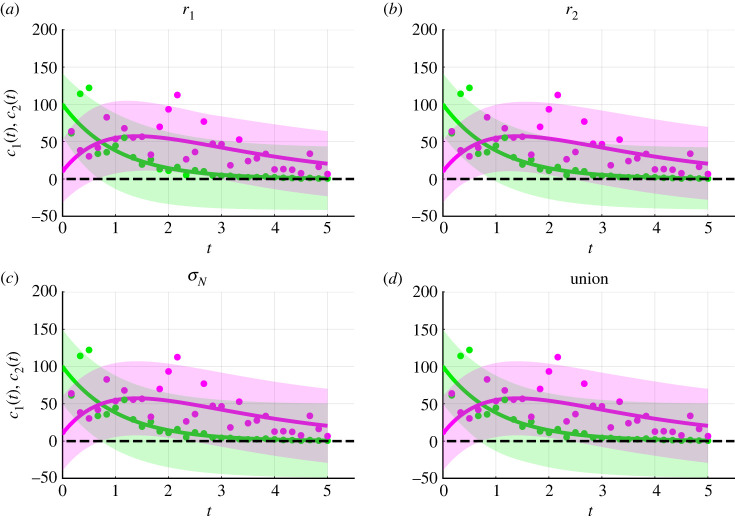
Bonferroni correction-based confidence sets for data realizations for the caricature ODE model with linear reactions (equation (3.1)) and intentional misspecification of the measurement error model. (*a*–*d*) Profile-wise confidence sets for data realizations (shaded) for (*a*) *r*_1_, (*b*) *r*_2_, (*c*) *σ*_N_ and (*d*) their union. Predictions suggest negative concentrations which are non-physical. The model, parameter values and colours are identical to figure 3. The black-dashed line corresponds to zero concentration; predictions below this level are not physically realistic.

We now generate a range of predictions. Profile-wise confidence sets for the mean reveal how uncertainty in estimates of mathematical model parameters, *r*_1_ and *r*_2_, results in uncertainty in predictions (figure 3*g*,*h*,*j*,*k*). For example, figure 3*g*,*j* shows that uncertainty in *r*_1_ results in greater uncertainty in *c*_2_(*t*) close to *t* = 1 as opposed to close to *t* = 0 and *t* = 5. By contrast, figure 3*h*,*k* shows that uncertainty in *r*_2_ results in greater uncertainty in *c*_2_(*t*) for *t* ≥ 1 than 0 < *t* < 1. In addition, we observe that uncertainty in *r*_1_ contributes to greater uncertainty in predictions for *c*_1_(*t*) than uncertainty in *r*_2_ (figure 3*g*,*h*). Taking the union of the profile-wise confidence sets for the model solution, we observe the overall uncertainty due to mathematical model parameters (figure 3*i*). Thus far these results appear to be physically realistic. However, now we consider Bonferroni correction-based profile-wise confidence sets for data realizations, and their union, that incorporate uncertainty in both the mathematical model parameters and measurement error model parameters (figure 4). These predictions of data realizations generate results with negative concentrations (figure 4). Such non-physical predictions are a direct consequence of using the additive Gaussian error model which suggests that this error model may not be appropriate in this situation.

**Figure 5.**
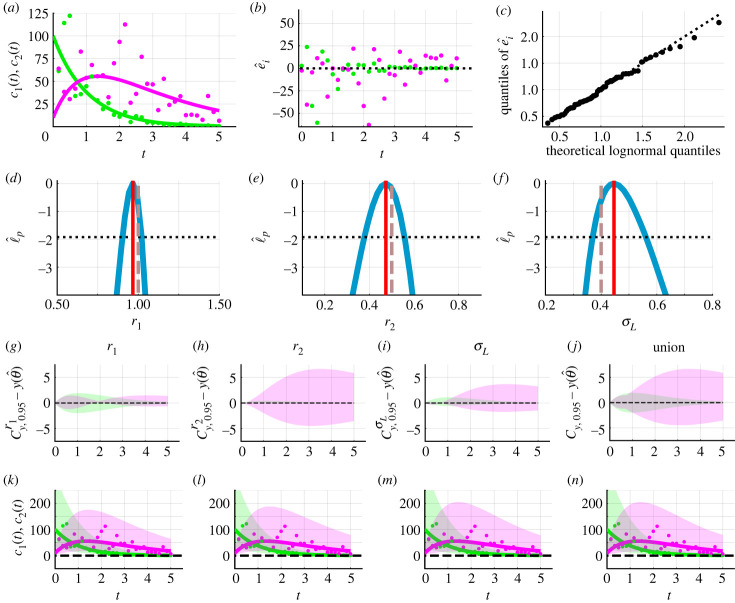
Caricature ODE model with linear reactions (equation (3.1)) and the correct model specification using the lognormal measurement error model. (*a*) Synthetic data (circles) at 31 equally spaced time points from *t* = 0.0 to *t* = 5.0 are generated by simulating equation (3.2), the lognormal measurement error model, known model parameters (*r*_1_, *r*_2_, *σ*_L_) = (1.0, 0.5, 0.4), and fixed initial conditions (*c*_1_(0), *c*_2_(0)) = (100.0, 10.0). Equation (3.2) simulated with the MLE (*r*_1_, *r*_2_, *σ*_L_) = (0.97, 0.47, 0.45) (solid). Throughout *c*_1_(*t*) (solid green) and *c*_2_(*t*) (solid magenta). (*b*) Difference between the observed data and the best-fit model solution, quantified through the ratios ${\hat{e}}_{i}=y_{i}^{o}/y_{i}(\hat{\theta })$. (*c*) Lognormal quantile–quantile plot of ratios ${\hat{e}}_{i}=y_{i}^{o}/y_{i}(\hat{\theta })$. (*d*–*f*) Profile log-likelihoods (blue) for (*d*) *r*_1_, (*e*) *r*_2_ and (*f*) *σ*_L_ with MLE (red-dashed), an approximate 95% confidence interval threshold (horizontal black-dashed) and known model parameters (vertical brown dashed). (*g*–*j*) Difference between Bonferroni correction-based confidence set for model solution and the solution of the mathematical model evaluated at the MLE for (*g*) *r*_1_, (*h*) *r*_2_, (*i*) *σ*_L_ and (*j*) their union. (*k*–*n*) Profile-wise Bonferroni correction-based confidence sets for model realizations (shaded) and the solution of the mathematical model evaluated at the MLE (solid line).

Re-analysing the data in figure 3*a* using the lognormal error model, we avoid any non-physical results. The MLE, $\hat{\theta }=(r_{1},r_{2},\sigma_{L})=(0.97,0.47,0.45)$, is close to the known values. The differences between the observed data and the best-fit model solution, quantified through the ratios ${\hat{e}}_{i}=y_{i}^{o}/y_{i}(\hat{\theta })$, are reasonably described by the lognormal distribution (figure 5*c*). Profile log-likelihoods suggest model parameters are practically identifiable (figure 5*d*–*f*). Approximate 95% confidence intervals, *r*_1_ ∈ (0.90, 1.02), *r*_2_ ∈ (0.38, 0.56) and *σ*_L_ ∈ (0.37, 0.56), capture known parameters and show that using the additive Gaussian error model overestimated uncertainty in *r*_1_. Profile-wise confidence sets for data realizations and their union are non-negative and so physically realistic (figure 5*k*–*n*). Electronic supplementary material, S6, presents additional quantile–quantile plots with and without misspecification of the measurement error model.

### 3.2. Temporal nonlinear models

It is straightforward to explore mathematical models of increasing complexity within the framework. A natural extension of equation (3.1) assumes that chemical reactions are rate-limited and nonlinear:

3.3 $$\begin{array}{rl} & \frac{dc_{1}(t)}{dt}=-\frac{V_{1}c_{1}(t)}{K_{1}+c_{1}(t)}\\ \;\mathrm{and}\; & \frac{dc_{2}(t)}{dt}=\frac{V_{1}c_{1}(t)}{K_{1}+c_{1}(t)}-\frac{V_{2}c_{2}(t)}{K_{2}+c_{2}(t)}. \end{array}\}$$

Here *V*_*i*_ and *K*_*i*_ represent maximum reaction rates and Michaelis–Menten constants for chemical species *C*_*i*_, with concentrations *c*_*i*_(*t*), for *i* = 1, 2. We solve equation (3.3) numerically. We treat the initial conditions *c*_1_(0) and *c*_2_(0) as known. Then equation (3.3) is characterized by four parameters *θ* = (*V*_1_, *K*_1_, *V*_2_, *K*_2_) that we will estimate. These four parameters are structurally identifiable. Note that the previous example, equation (3.1), only involved two mathematical model parameters and so our use of the profile log-likelihood in that case could have been avoided by working directly with the full likelihood; however, in this case, we have four unknown parameters in the mathematical model and so visual interpretation of the full likelihood is not straightforward. While one could marginalize the full likelihood for each parameter this often involves sampling-based integration methods that are typically more computationally expensive than optimization procedures that are required to obtain profile log-likelihoods for each parameter. Furthermore, working directly with the full likelihood to generate predictions can result in an order of magnitude increase in computational time in comparison to profile-wise predictions [27].

**Figure 6.**
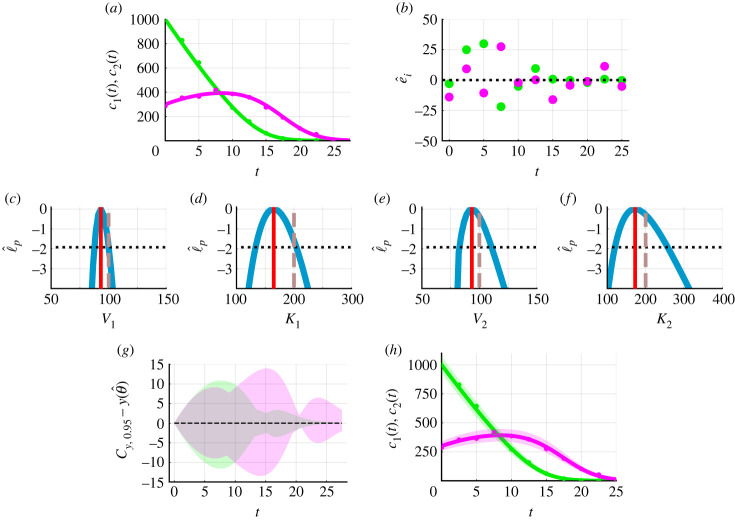
Caricature ODE model with nonlinear reaction terms (equation (3.3)) and the Poisson measurement error model. (*a*) Synthetic data (circles) at 11 equally spaced time points from *t* = 0.0 to *t* = 25.0 are generated by simulating equation (3.3), the Poisson measurement error model, known model parameters (*V*_1_, *K*_1_, *V*_2_, *K*_2_) = (100, 200, 100, 200), and fixed initial conditions (*c*_1_(0), *c*_2_(0)) = (1000, 300). Solution of equation (3.3) evaluated at the MLE (*V*_1_, *K*_1_, *V*_2_, *K*_2_) = (93.2, 164.8, 93.4, 172.7) (solid). Throughout *c*_1_(*t*) (solid green) and *c*_2_(*t*) (solid magenta). (*b*) Residuals ${\hat{e}}_{i}=y_{i}^{o}-y_{i}(\hat{\theta })$ with time *t*. (*c*–*f*) Profile log-likelihoods (blue) for (*c*) *V*_1_, (*d*) *K*_1_, (*e*) *V*_2_ and (*f*) *K*_2_ with MLE (red-dashed), an approximate 95% confidence interval threshold (horizontal black-dashed) and known model parameters (vertical brown dashed). (*g*) Difference between union of Bonferroni correction-based confidence sets for the model solution and the solution of the mathematical model evaluated at the MLE. (*h*) Union of Bonferroni correction-based profile-wise confidence sets for data realizations. Approximate 95% confidence intervals computed from profile log-likelihoods are *V*_1_ ∈ (87.7, 100.0), *K*_1_ ∈ (133.2, 204.0), *V*_2_ ∈ (82.6, 110.6) and *K*_2_ ∈ (120.1, 257.5). The MLE is $\hat{\theta }=(V_{1},K_{1},V_{2},K_{2})=(93.3,164.8,93.4,172.7)$.

We generate synthetic data using equation (3.3), the Poisson measurement error model, model parameters, *θ* = (*V*_1_, *K*_1_, *V*_2_, *K*_2_) = (100, 200, 100, 200), and initial conditions (*c*_1_(0), *c*_2_(0)) = (1000, 300) (figure 6*a*). Using equation (3.3) and the Poisson measurement error model, we seek estimates of *V*_1_, *K*_1_, *V*_2_ and *K*_2_ and generate predictions. Simulating the mathematical model with the MLE, we observe excellent agreement with the data (figure 6*a*). Profile log-likelihoods for *V*_1_, *K*_1_, *V*_2_ and *K*_2_ capture known parameter values and show that these parameters are practically identifiable. Predictions, in the form of the union of profile-wise confidence sets for the means (figure 6*g*) and the union of profile-wise confidence sets for realizations (figure 6*h*), show greater uncertainty at higher concentrations. Re-analysing these data using the additive Gaussian measurement error model results in non-physical predictions as we predict negative concentrations at later times where *c*_1_(*t*) and *c*_2_(*t*) are close to zero. The framework is straightforward to apply to other ODEs with nonlinear reaction terms, for example the Lotka–Volterra predator–prey model (electronic supplementary material, S4.1).

### 3.3. Spatio-temporal models

Throughout mathematical biology and ecology, we are often interested in dynamics that occur in space and time [18–22]. This gives rise to spatio-temporal data that we analyse with spatio-temporal models such as reaction–diffusion models. Reaction–diffusion models have been used to interpret a range of applications including chemical and biological pattern formation, spread of epidemics, and animal dispersion, invasion and interactions [18–22,70–72]. As a caricature example, consider a system of two diffusing chemical species in a spatial domain −∞ < *x* < ∞ subject to the reactions in equation (3.1). The governing system of PDEs is

3.4 $$\begin{array}{rl} & \frac{\partial c_{1}(t,x)}{\partial t}=D\frac{\partial^{2}c_{1}(t,x)}{\partial x^{2}}-r_{1}c_{1}(t,x))\\ \mathrm{and}\;\; & \frac{\partial c_{2}(t,x)}{\partial t}=D\frac{\partial^{2}c_{2}(t,x)}{\partial x^{2}}+r_{1}c_{1}(t,x)-r_{2}c_{2}(t,x). \end{array}\}$$

Here, *D* represents a constant diffusivity. We choose initial conditions to represent the release of chemical *C*_1_ from a confined region:

3.5 $$c_{1}(0,x)=\left\{ \begin{array}{cc} C_{0}\; & |x|<h,\;\\ 0\; & |x|>h,\; \end{array}\right.$$

and

3.6 $$c_{2}(0,x)=0,\;-\infty <x<\infty .$$

Solving equations (3.4)–(3.5) analytically, for *r*_1_ ≠ *r*_2_, gives (electronic supplementary material, S1) [73,74]

3.7 $$c_{1}(t,x)=\frac{C_{0}}{2}\left[\mathrm{erf}\left(\frac{h-x}{2\sqrt{Dt}}\right)+\mathrm{erf}\left(\frac{h+x}{2\sqrt{Dt}}\right)\right]\exp(-r_{1}t)$$

and

3.8 $$c_{2}(t,x)=\left(\frac{r_{1}}{r_{2}-r_{1}}\right)\frac{C_{0}}{2}\left[\mathrm{erf}\left(\frac{h-x}{2\sqrt{Dt}}\right)+\mathrm{erf}\left(\frac{h+x}{2\sqrt{Dt}}\right)\right]\left(\exp(-r_{1}t)-\exp(-r_{2}t)\right),$$

where $\mathrm{erf}(z)=2/\sqrt{\pi }\int_{0}^{z}\exp(\eta^{2})\;d\eta$ is the error function [74]. An analytical solution for the special case *r*_1_ = *r*_2_ can also be obtained and has a different format where again *c*_2_(*t*, *x*) is proportional to *c*_1_(*t*, *x*). Assuming that *C*_0_ and *h* are known, equations (3.7) and (3.8) are characterized by three unknown parameters (*D*, *r*_1_, *r*_2_).

**Figure 7.**
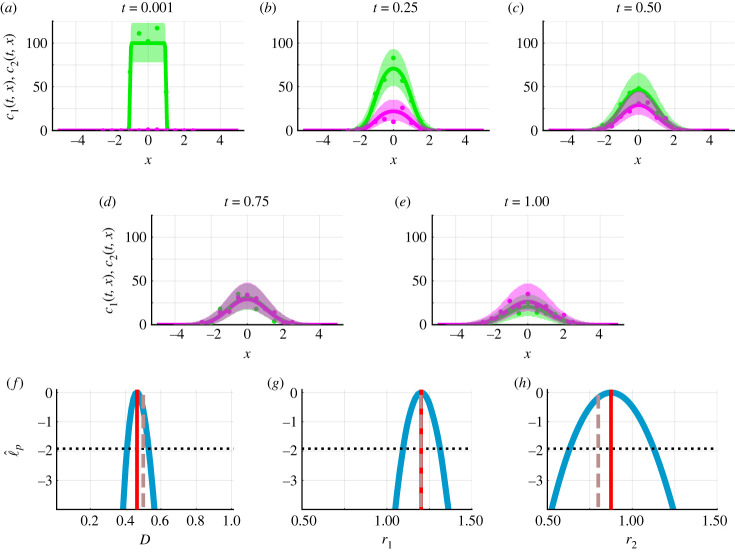
Caricature PDE model (equations (3.7) and (3.8)) with Poisson measurement error model. Synthetic data (circles) generated by solving equations (3.7) and (3.8) and the Poisson measurement error model with known model parameters *θ* = (*D*, *r*_1_, *r*_2_) = (0.5, 1.2, 0.8). (*a*–*e*) Union of Bonferroni correction-based profile-wise confidence sets for data realizations of *c*_1_(*t*) (green shaded) and *c*_2_(*t*) (magenta shaded) for (*a*) *t* = 0.001, (*b*) *t* = 0.25, (*c*) *t* = 0.50, (*d*) *t* = 0.75 and (*e*) *t* = 1.0. Data points are measured at 11 equally spaced positions between *x* = −2.5 and *x* = 2.5, inclusive. The solution of equations (3.7) and (3.8) evaluated at the MLE shown for *c*_1_(*t*) (green solid) and *c*_2_(*t*) (magenta solid). (*f*–*h*) Profile log-likelihoods (blue) for (*f*) *D*, (*g*) *r*_1_ and (*h*) *r*_2_ with MLE (red-dashed), an approximate 95% confidence interval threshold (horizontal black-dashed) and known model parameters (vertical brown dashed).

We generate synthetic spatio-temporal data at eleven spatial points and five different times (figure 7*a*–*e*). To generate the synthetic data, we use equations (3.7) and (3.8), the Poisson measurement error model, and set *θ* = (*D*, *r*_1_, *r*_2_) = (0.5, 1.2, 0.8) and fix (*C*_0_(0), *h*) = (100, 1). To obtain estimates of *D*, *r*_1_, *r*_2_ and generate predictions, we use equations (3.7) and (3.8) and the Poisson measurement error model. Simulating the mathematical model with the MLE, we observe excellent agreement with the data (figure 7*a*–*f*). Univariate profile log-likelihoods for *D*, *r*_1_ and *r*_2_ are well-formed, capture the known parameter values, and suggest that these parameters are practically identifiable. Predictions, in the form of the union of profile-wise confidence sets for realizations (figure 6*h*), show that there is greater uncertainty at higher chemical concentrations. This framework also applies to systems of PDEs that are solved numerically (electronic supplementary material, S2). Previous comments exploring measurement error model misspecification for systems of ODEs also hold for systems of PDEs.

## 4. Coverage

Frequentist methods for estimation, identifiability and prediction are generally concerned with constructing estimation procedures with reliability guarantees, such as coverage of confidence intervals and sets. For completeness, we explore coverage properties numerically. We present an illustrative example revisiting equation (3.1) with the additive Gaussian noise model and now fix *σ*_*N*_ = 5. This results in a model with two parameters, *θ* = (*r*_1_, *r*_2_) = (1.0, 0.5), that we estimate. Initial conditions (*c*_1_(0), *c*_2_(0)) = (100, 25) are fixed. The same evaluation procedure can be used to assess coverage properties for non-Gaussian noise models, such as the lognormal error model (electronic supplementary material, S5).

We generate 5000 synthetic datasets using the same mathematical model, measurement error model and model parameters, *θ*. Each dataset comprises measurements of *c*_1_(*t*) and *c*_2_(*t*) at 16 equally spaced time points from *t* = 0.0 to *t* = 2.0. For each dataset, we compute a univariate profile log-likelihood for *r*_1_ and use this to form an approximate 95.0% confidence interval for *r*_1_. We then test whether this approximate 95.0% confidence interval contains the true value of *r*_1_. This holds for 95.2% of the datasets, corresponding to an observed coverage probability of 0.952. Similarly, the observed coverage probability for *r*_2_ is 0.946. Therefore, the observed coverage probabilities for both *r*_1_ and *r*_2_ are close to the target coverage probability of 0.950. In contrast to our profile-wise coverage approach, a full likelihood-based approach recovers an observed coverage probability of 0.950 for the confidence region for *r*_1_ and *r*_2_ (electronic supplementary material, S3).

For each dataset, we propagate forward variability in *r*_1_ to generate an approximate 95% confidence set for the model solution, $C_{y,0.95}^{r_{1}}$. We consider coverage of this confidence set from two perspectives. First, we explore coverage from the perspective of testing whether or not the true model solution, *y*(*t*; *θ*), is entirely contained within the confidence set and refer to this as *curvewise coverage*. Second, we discretize the model solution and for each point of the model solution, *y*(*t*_*i*_; *θ*) for *i* = 1, 2, 3, …, *N*, we test whether or not it is contained within the confidence set for the model solution and refer to this as *pointwise coverage*. Note that the time points at which we discretize the model solution do not need to be the same time points where measurements are observed. Previous profile likelihood-based methods focus only on pointwise predictions [56–58]. In our framework, curvewise properties are natural for model trajectories since we are interested in the variability of model solutions obtained by propagating forward variability in model parameters using a continuous deterministic mathematical model. Curvewise coverage properties are more challenging to achieve in general and pointwise coverage properties can help to explain why.

### 4.1. Curvewise coverage

**Figure 8.**
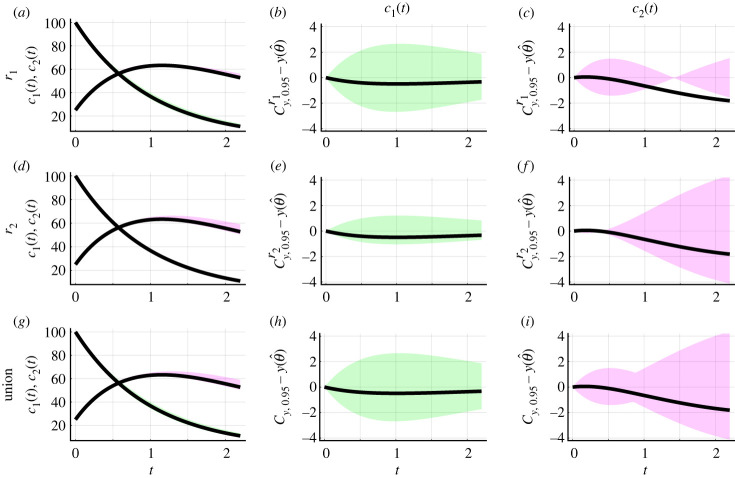
Curvewise confidence sets for the model solutions of a caricature ODE model with linear reaction terms (equation 3.1) and the additive Gaussian measurement error model with known *σ*_*N*_. (*a*) Confidence sets for model solution generated from uncertainty in *r*_1_, $C_{y,0.95}^{r_{1}}$ (shaded), and the true model solution, *y*(*θ*) (black). (*b*,*c*) Difference between curvewise confidence set and solution of the mathematical model evaluated at the MLE, $C_{y,0.95}^{r_{1}}-y(\hat{\theta })$ (*c*_1_(*t*) (shaded green) and *c*_2_(*t*) (shaded magenta)) and the difference between the true model solution and the solution of the mathematical model evaluated at the MLE, $y(\theta )-y(\hat{\theta })$ (black). (*d*–*f*) Results based on uncertainty in *r*_2_. (*g*–*i*) Results for the union of curvewise confidence sets. Throughout, to plot *y*(*θ*) the temporal domain is discretized into 100 equally spaced points (0.022 ≤ *t* ≤ 2.200) connected using a solid line.

For the problems we consider, variation in the confidence set at each time point is narrow relative to the overall variation in *c*_1_(*t*) and *c*_2_(*t*) over time (figure 8*a*). Therefore, we plot and examine the difference between the confidence set and the model solution at the MLE, $C_{y,0.95}^{r_{1}}-y(\hat{\theta })$, and the difference between the true model solution and the model solution at the MLE, $y(\theta )-y(\hat{\theta })$ (figure 8*b*,*c*). The *c*_1_(*t*) component of the true model solution, *y*(*t*; *θ*), is contained within the confidence set (figure 8*b*). However, the true model solution is only contained within the *c*_2_(*t*) component of the confidence set for *t* ≤ 1.056 (figure 8*c*). Hence, the true model solution is not contained within the confidence set $C_{y,0.95}^{r_{1}}$. We repeat this analysis for the confidence set $C_{y,0.95}^{r_{2}}$ (figure 8*d*–*f*) and the union of the confidence sets $C_{y,0.95}=C_{y,0.95}^{r_{1}}\cup C_{y,0.95}^{r_{2}}$ (figure 8*g*–*i*). By construction, the confidence set *C*_*y*,0.95_ has coverage properties that are at least as good as $C_{y,0.95}^{r_{1}}$ and $C_{y,0.95}^{r_{2}}$. For example, in figure 8*h*,*i,* the true model solution is contained within *C*_*y*,0.95_ whereas it is not contained within $C_{y,0.95}^{r_{1}}$. Assessing whether the model solution is or is not entirely contained within the confidence sets $C_{y,0.95}^{r_{1}}$, $C_{y,0.95}^{r_{2}}$ and *C*_*y*,0.95_ for each of the 5000 datasets, we obtain observed curvewise coverage probabilities of 0.007, 0.018 and 0.609, respectively. These observed coverage probabilities are much lower than results for confidence intervals of model parameters. However, in contrast to our profile-wise coverage results, a full likelihood-based approach recovers an observed curvewise coverage probability of 0.956 for the confidence set for model solutions (electronic supplementary material, S3).

Given the drastic differences in observed curvewise coverage probabilities between the profile likelihood-based method and full likelihood-based method one may expect that the confidence sets from the two methods are qualitatively very different. However, comparing the two confidence sets they appear to be qualitatively very similar (electronic supplementary material, S3). This suggests that subtle differences in confidence sets may play an important role in observed curvewise coverage probabilities. Full likelihood-based approaches are computationally expensive relative to profile likelihood-based methods, especially for models with many parameters. Here, we have only considered univariate profiles. However, an interesting approach is to use profile likelihood-based methods with higher-dimensional interest parameters. These have been shown to improve coverage properties relative to scalar valued interest parameters at a reduced computational expense relative to full likelihood-based methods [27].

### 4.2. Pointwise coverage

**Figure 9.**
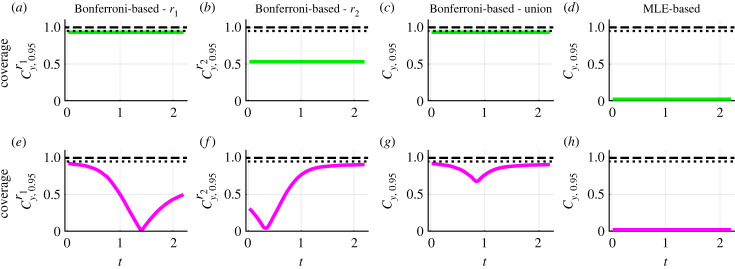
Pointwise coverage analysis for confidence sets for model solutions. Analysis performed using the caricature ODE model (equation (3.1)) as an illustrative example. (*a*–*h*) Pointwise coverage analysis of confidence sets for model solutions. Results for Bonferroni correction-based confidence sets for *r*_1_, *r*_2_, and their union are shown in (*a*,*e*), (*b*,*f*) and (*c*,*g*), respectively. Results for MLE-based confidence sets are shown in (*d*,*h*). The temporal domain is discretized into 100 equally spaced points (0.022 ≤ *t* ≤ 2.200). Horizontal dotted and horizontal dashed lines correspond to observed probabilities of 0.95 and 1.00, respectively.

Assessing pointwise coverage can help diagnose why we do not reach target curvewise coverage properties when propagating univariate profiles. This kind of diagnostic tool can be used to inform experimental design questions regarding when, and/or where, to collect additional data. In this context, the confidence sets can be interpreted as tools for sensitivity analysis. We discretize the temporal domain into 100 equally spaced points (0.022 ≤ *t* ≤ 2.200), and exclude *t* = 0 because initial conditions are treated as fixed quantities in this instance. For each dataset, time point, chemical concentration and confidence set, we test whether the true model solution is contained within the confidence set. For the component of the confidence set $C_{y,0.95}^{r_{1}}-y(\hat{\theta })$ concerning *c*_1_(*t*), the observed pointwise coverage is constant throughout time and equal to 0.932 which is relatively close to the desired value (figure 9*a*). By contrast, for the component of the confidence set $C_{y,0.95}^{r_{1}}-y(\hat{\theta })$ concerning *c*_2_(*t*), the observed pointwise coverage is initially equal to 0.920 at *t* = 0.022, then decreases over time reaching a minimal value of 0.012 at *t* = 1.408 before increasing to 0.497 at *t* = 2.200 (figure 9*e*). Similar behaviour is observed for the confidence set $C_{y,0.95}^{r_{2}}-y(\hat{\theta })$ (figure 9*b*,*f*). Taking the union of the confidence sets, we obtain more conservative confidence sets, with an observed pointwise coverage for *c*_1_(*t*) of 0.932 throughout (figure 9*c*) and an observed pointwise coverage for *c*_2_(*t*) of at least 0.681 (figure 9*g*). Note that the solution of the mathematical model evaluated at the MLE, $y(t;\hat{\theta })$, is not identical to the true model solution so, as expected, the observed pointwise coverage probability of this single trajectory is zero at all time points (figure 9*d*,*h*).

**Figure 10.**
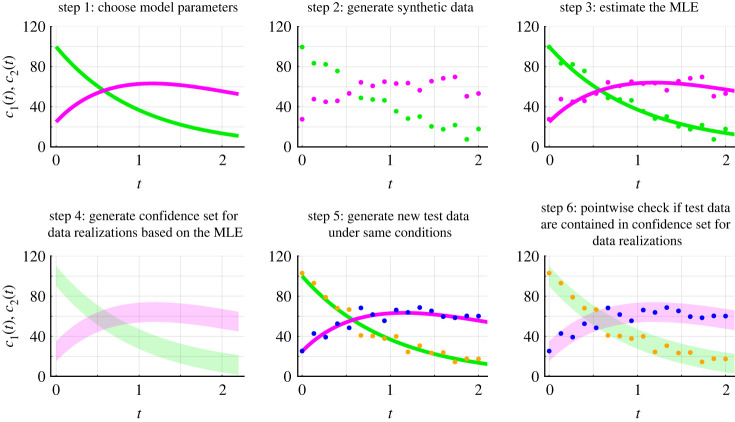
Schematic for evaluation procedure used to test coverage properties of confidence sets for model realizations. In this work, we repeat these steps 5000 times. Example presented using the MLE-based approach, and is readily adapted for Bonferroni correction-based confidence sets for model realizations by modifying step four.

**Figure 11.**
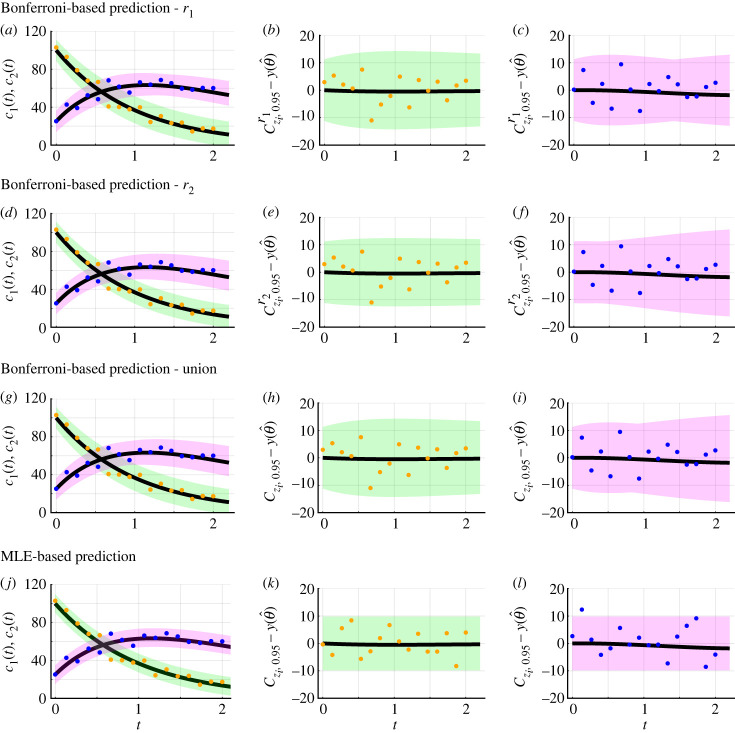
Confidence sets for model realizations of a caricature ODE model with linear reaction terms (equation 3.1) and the additive Gaussian measurement error model with known *σ*_*N*_. (*a*) Bonferroni correction-based confidence set for *r*_1_. (*b*,*c*) Difference between the Bonferroni correction-based confidence set for *r*_1_ and solution of the mathematical model evaluated at the MLE, $C_{y,0.95}^{r_{1}}-y(\hat{\theta })$ (*c*_1_(*t*) (shaded green) and *c*_2_(*t*) (shaded magenta)) and the difference between the true model solution and the solution of the mathematical model evaluated at the MLE, $y(\theta )-y(\hat{\theta })$ (black). (*d*–*i*) Results for Bonferroni correction-based confidence sets for (*d*–*f*) *r*_2_ and (*g*–*i*) the union. (*j*–*l*) Results for MLE-based confidence set.

We now explore MLE-based and Bonferroni correction-based confidence sets for model realizations in a pointwise manner. For both methods, we apply the same evaluation procedure (figure 10). For each of the 5000 synthetic datasets, we generate the confidence set for the data realizations and then generate a new synthetic dataset under the same conditions as the original synthetic dataset. In particular, the new dataset is generated at the same time points using the same mathematical model, measurement error model and parameter values. This approach can be thought of as a test of the predictions under replication of the experiment. For each new data point, which includes fifteen equally spaced data points from *t* = 0.13 to *t* = 2.00, we test whether or not it is contained within the confidence set for the model realization. Results for a single synthetic dataset show that Bonferroni correction-based confidence sets for model realizations based on *r*_1_ (figure 11*a*–*c*), *r*_2_ (figure 11*d*–*f*) and their union (figure 11*g*–*i*) can overcover relative to the MLE-based approach (figure 11*j*–*l*).

**Figure 12.**
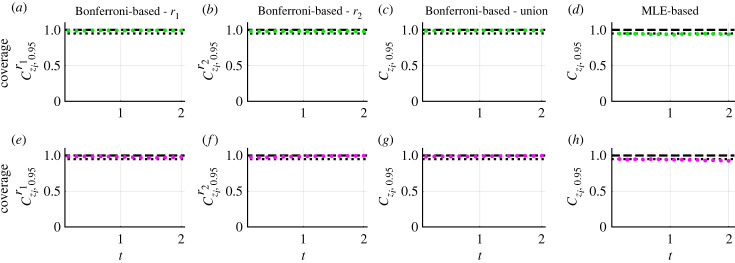
Pointwise coverage analysis for confidence sets for model realizations. Analysis performed using the caricature ODE model (equation (3.1)) as an illustrative example. (*a*–*h*) Pointwise coverage of confidence sets for model realizations. Results for Bonferroni correction-based confidence sets for *r*_1_, *r*_2_, and their union are shown in (*a*,*e*), (*b*,*f*) and (*c*,*g*), respectively. Results for MLE-based confidence sets are shown in (*d*,*h*). Horizontal dotted and horizontal dashed lines correspond to observed probabilities of 0.95 and 1.00, respectively.

Analysing results for the 5000 synthetic datasets, we find that the average observed pointwise coverage probability for the MLE-based confidence set for model realizations across all time points and the two chemical species is 0.937. Pointwise coverage properties per time point and chemical species for the MLE-based approach are shown in figure 12*d*,*h*. In this example, statistical noise is large relative to the difference in the true model solution and the solution of the mathematical model evaluated at the MLE, $y(t;\theta )-y(t;\hat{\theta })$, such that the coverage properties are relatively close to the target coverage probability of 0.950. The average pointwise coverage for the Bonferroni correction-based confidence set for model realizations is 0.985 for *r*_1_, 0.982 for *r*_2_ and 0.990 for their union. Pointwise coverage properties per time point and chemical species for the Bonferroni correction-based approaches are shown in figure 12*a*–*c*,*e*–*g*. For this particular example, the Bonferroni correction-based value consistently exceeds the target coverage probability. Using a full likelihood-based method recovers an observed average pointwise coverage probability 0.994 for the Bonferroni correction-based confidence set for model realizations (electronic supplementary material, S3). Note that since the MLE-based confidence set for model realizations depends only on the MLE, the confidence set is independent of whether a profile likelihood-based or full likelihood-based approach is implemented.

While the framework presented in this section is straightforward to apply to other mathematical models and measurement error models, coverage properties should be interpreted and assessed on a case-by-case basis. In electronic supplementary material, S5, we present such an example using the lognormal measurement error model and find similar results to those discussed here. Other frequentist evaluation procedures can also be used to explore coverage properties of confidence sets for model realizations. For example, for a dataset with *I* elements, we could generate a confidence set for model realizations based on the first *k* < *I* time points of data and then test if one, or more, of the remaining *I* − *k* elements of the dataset are contained in the confidence set.

## 5. Conclusion

In this review, we demonstrate how to practically implement a variety of measurement error models in a general profile likelihood-based framework for parameter estimation, identifiability analysis and prediction. Illustrative case studies explore additive, multiplicative, discrete, and continuous measurement error models and deal with the commonly encountered situation of noisy and incomplete data. Mathematical models in the case studies are motivated by the types of models commonly found in the systems biology literature and the mathematical biology literature. Within the framework, assessing uncertainties in parameter estimates and propagating forward these uncertainties to form predictions allow us to assess the appropriateness of measurement error models and make direct comparisons to data. Furthermore, techniques to assess pointwise and curvewise coverage properties provide useful tools for experimental design and sensitivity analysis. The profile likelihood-based methods, based on numerical optimization procedures, are computationally efficient and a useful approximation to full likelihood-based methods (electronic supplementary material, S3) [27]. Open source Julia code to reproduce results is freely available on GitHub (https://github.com/ryanmurphy42/Murphy2023ErrorModels). These implementations can be adapted to deal with other forms of mathematical models or they could be adapted for implementation within other software frameworks; however, we prefer Julia because it is freely available and computationally efficient.

We illustrate the framework using simple caricature models to emphasize the practical implementation of the methods and how to interpret results, rather than the details of each mathematical model. This includes systems of ODEs that are often used in the systems biology literature (§§3.1 and 3.2; electronic supplementary material, S4) and systems of PDEs routinely used in the mathematical biology literature (§3.3). ODE-based models are also routinely used to describe biological population dynamics [75] and disease transmission [76]. As parameter estimation, identifiability analysis, and prediction within the profile likelihood-based framework depend only on the solution of the mathematical model, the solution can be obtained analytically or numerically. Analytical solutions are preferred over numerical solutions for computational efficiency; however, closed-form exact solutions cannot always be found. For this reason, we implement a number of case studies that involve working with simple exact solutions, as well as working with numerical solutions obtained using standard discretizations of the governing differential equations. One can also consider other mathematical models with the framework, such as difference equations that are often used in applications about ecology (electronic supplementary material, S4) [20,23–26]. More broadly the framework can apply to stochastic differential equation-based models [77] and stochastic simulation-based models [5,78–81]. Extensions to models that incorporate process noise are of interest [26,82–86].

The framework is well suited to consider a variety of measurement error models. Illustrative case studies explore the additive Gaussian error model, the multiplicative lognormal model and the discrete Poisson model. All example calculations presented in this review take an approach where synthetic data are generated using a mathematical model rather than working with experimental measurements. This is a deliberate choice that allows us to explicitly explore questions of model misspecification and model choice unambiguously since we have complete control of the underlying data generating process. By definition, samples from the lognormal distribution are positive so we deliberately avoid situations where the observed data are zero when using the lognormal measurement error model. A different error model should be considered in such a case, for example, based on the zero-modified lognormal distribution [62,87]. For both the lognormal and Poisson error models, we also avoid situations where the observed data are positive and the model solution is identically zero. For example, our solutions of ODE-based models approach zero at late time but remain positive for all time considered in this work. Exploring error models for reaction–diffusion PDEs with nonlinear diffusion is of interest, for example those that give rise to travelling wave solutions describing biological invasion with sharp boundaries [88–90]. In such an example, we may expect to evaluate the error model, and so the likelihood function, at points in space where the data are positive but the model solution is zero. How to handle such a situation and which measurement error model to incorporate is an interesting question that could be explored by extending the tools developed in this review.

Within the framework one could also consider other forms of multiplicative error models, for example based on the gamma distribution [8,9], of which the exponential and Erlang distributions are special cases, or based on the beta distribution [26]. A different form of the lognormal distribution with mean equal to *y*_*i*_(*θ*) could also be considered within the framework and is given by $y_{i}|\theta ∼\mathrm{LogNormal}(\log(y_{i}(\theta ))-\sigma_{L}^{2}/2,\sigma_{L})$. Multiplicative noise can be also be implemented in other forms. We have considered multiplicative noise of the form $y_{i}^{o}=y_{i}(\theta )\eta_{i}$ with $\eta_{i}∼\mathrm{LogNormal}(0,\sigma_{L}^{2})$ (equation (2.4)), which for a straight line model, *y*(*θ*) = *c* + *mx*, would be $y_{i}^{o}=(c+mx_{i})\eta_{i}$. However, multiplicative noise could also be associated with a component of the model solution. As a specific example from a protein quantification study [11] consider the straight line model where multiplicative noise is incorporated into the slope of the equation but not the *y*-intercept, i.e $y_{i}^{o}=c+mx_{i}\eta_{i}$ with $\eta_{i}∼\mathrm{LogNormal}(0,\sigma_{L}^{2})$. One could also relax assumptions in the Poisson distribution that the variance is equal to the mean, in which case the negative binomial distribution may be useful [84]. The framework also applies to other discrete distributions such as the binomial model [91,92]. Different measurement error models could also be studied for example the proportional, exponential, and combined additive and proportional error models that are used in pharmacokinetic modelling [93]. Throughout we assume that errors are independent and identically distributed. Extending the noise model to consider correlated errors is also of interest [94,95]. Assessing coverage properties using different evaluation procedures and assessing predictive capability through the lens of tolerance intervals is also of interest [68,96]. Overall, the choice of which mathematical model and measurement error model to use should be considered on a case-by-case basis and can be explored within this framework.

## Data Availability

Code to reproduce all results in the paper is freely and publicly available from the Zenodo repository: https://zenodo.org/records/10452931 [97]. Supplementary material is available online [98].

## Appendix A. Code

Julia implementations of all computations are available on GitHub (https://github.com/ryanmurphy42/Murphy2023ErrorModels). Here, we highlight key packages and code used in our implementation.

Throughout we assess structural identifiability using the StructuralIdentifiability package [39]. To estimate parameters and explore practical identifiability using profile log-likelihoods, we find that it is straightforward to compute the log-likelihood for a range of error models using the loglikelihood function in the Distributions package [97]. For example, we evaluate the log-likelihood for the additive Gaussian, multiplicative lognormal and discrete Poisson measurement error models using loglikelihood$\left(\mathrm{Normal}(y_{i}(\theta ),\sigma^{2}),y_{i}^{o}\right)$, loglikelihood$\left(\mathrm{LogNormal}(\log(y_{i}(\theta )),\sigma_{L}^{2}),y_{i}^{o}\right)$ and loglikelihood$\left(\mathrm{Poisson}(y_{i}(\theta )),y_{i}^{o}\right)$, respectively. Approximate confidence interval thresholds are obtained computationally by c=quantile(Chisq(*ν*), 1 − *α*)/2, using the Distributions package [97]. For example, 90%, 95%, 99% and 99.9% (*α* = 0.100, 0.050, 0.010, 0.001, respectively) correspond to threshold values of $\ell_{c}=-1.35,\;-1.92,\;-3.32\;\;\mathrm{and}-5.51$, respectively [40,67].

All systems of differential equations are solved numerically using the default ODEproblem solver in the DifferentialEquations package [99]. To perform numerical maximization, we find that the Nelder–Mead local optimization routine, with default stopping criteria, within the NLopt optimization package performs well for the problems in this study [100].
